# *Artemisia argyi* water extract promotes selenium uptake of peach seedlings

**DOI:** 10.3389/fpls.2022.1014454

**Published:** 2022-11-10

**Authors:** Lijin Lin, Jiangyue Wang, Ming’an Liao, Rongping Hu, Qunxian Deng, Zhihui Wang, Xun Wang, Yi Tang

**Affiliations:** ^1^ Institute of Pomology and Olericulture, Sichuan Agricultural University, Chengdu, China; ^2^ College of Horticulture, Sichuan Agricultural University, Chengdu, China; ^3^ Institute of Sichuan Edible Fungi, Chengdu, China

**Keywords:** allelopathy, fruit tree, plant physiology, selenium transportation, growth

## Abstract

Soil in most areas of the world is selenium (Se) deficient, which results a low Se content in agricultural products. To improve the fruit tree Se accumulation, the effects of different *Artemisia argyi* water extract concentrations (0, 100, 200, 300, and 400-fold dilutions) on the growth and Se accumulation of peach seedlings were studied by a pot experiment. A 300- and 400-fold dilution of *A. argyi* water extract increased the root and shoot biomass (dry weight), leaf chlorophyll *a* content, superoxide dismutase (SOD) activity, and peroxidase (POD) activity of peach seedlings, but decreased the leaf chlorophyll *a*/*b*. Different *A. argyi* water extract concentrations had no significant effects on peach leaf chlorophyll *a* content of peach seedlings, but increased the leaf carotenoid content, catalase (CAT) activity, and soluble protein content. Different *A. argyi* water extract concentrations increased the total Se, inorganic Se, and organic contents in roots and shoots of peach seedlings to some extent. Furthermore, *A. argyi* water extract concentration exhibited a linear relationship with the root and shoot total Se contents. Compared with the control, the 100-, 200-, 300-, and 400-fold dilutions of *A. argyi* water extract increased the shoot total Se content by 18.95%, 31.31%, 39.32%, and 51.59%, respectively. Different *A. argyi* water extract concentrations also increased the leaf Se metabolism-related enzyme activities of peach seedlings, including the activities of adenosine triphosphate sulfurase (ATPS), adenosine 5’-phosphosulfate reductase (APR), and serine acetyltransferase (SAT), as well as selenocysteine methyltransferase (SMT) to some extent. Moreover, correlation and grey relational analyses revealed the root total Se content, CAT activity, and ATPS activity to be closely associated with the total shoot Se content. Therefore, applying *A. argyi* water extract can thus promote the growth and Se uptake of peach seedlings, and the future study should focus on the application effects of Se uptake in peach fruits.

## Introduction

Selenium (Se) is an essential trace element in the human body and is extremely important for human health, having anti-cancer, anti-oxidation, and immunity enhancing functions. Se deficiency in the human body causes diseases such as Keshan disease and Kashin-Beck disease (Zhang et al., 2007; Qu et al., 2010; Méplan and Hesketh, 2012). Most areas in the world are Se-deficient, resulting in an extremely low Se content in agricultural products (Sun et al., 2017). The Se in humans and animals mainly derive from crops, while crop Se mainly derives from the soil; crops can convert inorganic Se into organic Se after being absorbed from the soil (Chen et al., 2017; Natasha et al., 2018). So, crop Se sources are the best option for human Se supplementation, and screening out methods to improve crop (especially fruit) Se uptake are urgently needed.

External Se fertilizer application is mostly used to increase crop Se content, but the continuous use or excessive application of Se fertilizer can easily become toxic for crops (Mostofa et al., 2017). Crop straw returning is an agronomic measure commonly used in agricultural production, which can increase crop yield and improve crop quality, because crop straw contains nitrogen (N), phosphorus (P), and potassium (K), as well as other elements and organic matter, to improve soil fertility (Lu and Xie, 2011; Li et al., 2018; Qin, 2021). Moreover, crop straw can release the allelochemicals during decay, which may produce the allelopathy to affect the growth and nutrient absorption of other crop (Ghnaya et al., 2016). The allelopathy of crop straw also affects the heavy metal accumulation in crops (Gu et al., 2017; Liu et al., 2018a). Under cadmium (Cd) contaminated soil, crop straw decreases the Cd accumulation in other crops by effectively reducing soil available Cd concentration (Wang et al., 2019; Lian et al., 2020). When peanut, rice, and maize straws are returned to the field, different wheat growth effects and Cd accumulation occur. Peanut straw decreases wheat Cd content, while increasing arsenic (As) uptake; rice straw does not significantly affect wheat Cd content, but decreases As uptake; maize straw decreases both Cd and As uptake in wheat (Cao et al., 2019). Other studies on heavy metal hyperaccumulators and accumulators, and fruit trees (Lin et al., 2014; Lin et al., 2015; He et al., 2017) have shown similar effects to wheat. These results may be related to the different allelochemicals released from the various straws, which may activate or complex the heavy metal in soil (Lin et al., 2015; Cao et al., 2019; Wang et al., 2019). For Se uptake, *Pterocypsela laciniata* straw application promotes grape seedling Se uptake, but inhibits grape seedling growth to a certain extent (Liu et al., 2021), which may be related to the crop straw increasing soil selenite bioavailability by activating soil Se (Wang, 2019a). However, no other reports exist regarding crop straw returning effects on other crop Se uptake. Thus, crop straw returning may affect plant Se uptake, but this requires screening of more crop straw and living crop combinations.

Peach (*Prunus persica*) is a global distribution fruit tree with delicious fruits. Its main rootstock materials are wild peach (*P. persica*) or mountain peach (*P. davidiana*) (Yang et al., 2011). *Artemisia argyi* is a perennial herb used for medicine, food, and dyes, as well as many other uses. There main components of *A. argyi* extract are terpenes, flavonoids, volatile oils, and trace elements, which can produce the allelopathy on the other crops (Li et al., 2016). If *A. argyi* extract is applied to wild peach (rootstock material), its growth and Se uptake may be promoted, and the improvement of Se contents in peach fruits also can improve its commercial value (Zhang, 2011). Therefore, in this experiment, different *A. argyi* water extract concentration effects on the growth and Se accumulation characteristics of peach seedlings were studied. The aim of this study was to investigate whether *A. argyi* water extract could promote the growth and Se uptake of peach seedlings, and to determine the best *A. argyi* water extract concentration for Se-enriched peach production.

## Materials and methods

### Materials

Fluvo-aquic soil was collected from a farmland near the Chengdu Campus of the Sichuan Agricultural University (30°42′N, 103°51′E). Its basic physicochemical properties have previously been described (Liu et al., 2022).

The shoots of *A. argyi* were also collected from a farmland near the Chengdu Campus of the Sichuan Agricultural University in May 2021. These shoots were dried in a drying oven at 75°C until constant weight, and cut into pieces about 1 cm in length. The preparation method of *A. argyi* water extract was according to the boiling method of traditional Chinese medicine. A total of 10 g dried *A. argyi* pieces were added to 1500 mL distilled water, boiled, and then simmered on a low heat for 30 min. After cooling, the *A. argyi* water extract was added to a final volume of 1000 mL (the water was evaporated about 600 mL during heating), resulting in an *A. argyi*:water ratio of 1:100 (w/v); this solution was stored in a -18°C refrigerator until required.

Wild peach seeds were collected from a 5-year-old peach tree on a farm near the Chengdu Campus of the Sichuan Agricultural University in September 2021. The seeds were stored in moist sand until germination. In March 2022, the germinated seeds were sown into plug trays (50 holes) filled with perlite for seedling culturing. The plug trays were irrigated with Hoagland’s nutrient solution every three days. One month later, the peach seedlings were transplanted into pots when they grew to 10 cm in height.

### Experimental design

The experiment was conducted at a canopy of the Chengdu Campus of Sichuan Agricultural University from March to May 2022. In March 2022, each plastic pot (21 cm diameter and 20 cm depth) was filled with 3.0 kg of air-dried and crushed soil. Analytically pure Na_2_SeO_3_ was added and mixed into the soil to make a final soil Se concentration of 5 mg kg^−1^ (Hu et al., 2019). Each pot’s soil was watered every day to make sure that its soil moisture content was kept at 80% field capacity. In April 2022, four uniform peach seedlings (10 cm in height) from the plug trays cultured seedlings were transplanted into each pot, and evenly distributed in all four directions. Hereafter, different *A. argyi* water extract concentrations (0, 100, 200, 300, and 400-fold dilutions) were used to irrigate the pots. Each pot was irrigated 50 mL *A. argyi* water extract, and each treatment was repeated in triplicate (three pots) using a completely randomized design. Hereafter, pots were irrigated every seven days for a total of four irrigations. The pots were watered with tap water every day to maintain an adequate water supply for plant growth.

### Determination of indicators

One month after the initial *A. argyi* water extract irrigation (May 2022), mature leaves, each at the same position (middle part of the peach stem) were collected to determine the contents of photosynthetic pigments (chlorophyll *a*, chlorophyll *b*, and carotenoids), antioxidant enzyme [superoxide dismutase (SOD), catalase (CAT), and peroxidase (POD)] activities, soluble protein content, and Se metabolism-related enzyme [adenosine triphosphate sulfurase (ATPS), adenosine 5’-phosphosulfate reductase (APR), serine acetyltransferase (SAT), and selenocysteine methyltransferase (SMT)] activities. Photosynthetic pigment contents were extracted using the acetone-ethanol extraction method and determined at 663, 645, and 470 nm wavelengths using a spectrophotometer (Summit, Shanghai, China) according to Hao et al. (2004), and the chlorophyll *a*/*b* was calculated as the chlorophyll *a* content/chlorophyll *b* content. SOD, CAT, and POD activities were determined using the nitrotetrazole chloride reduction, potassium permanganate titration, and guaiacol methods, respectively, as described by Lin et al. (2020) and Hao et al. (2004). The Coomassie brilliant blue method was used for soluble protein content determination according to Hao et al. (2004). The activities of Se metabolism-related enzymes were determined using the enzyme linked immunosorbent assay (ELISA) kits (Shanghai Enzyme Link Biotechnology Co., Ltd., Shanghai, China) following the manufacturer’s instructions. Hereafter, whole plants were harvested and treated following the methods described by Li et al. (2022). Roots and shoots were divided, and their dry weights (biomass) were measured using an electronic balance. The dried samples were finely ground and digested with nitrate and perchloric acids, and reduced in hydrochloric acid to determine total Se content. Total Se content was subsequently measured using hydride generation-atomic fluorescence spectrometry (AFS-9700, Beijing Haiguang Instrument Co., Ltd., Beijing, China), as described by Li et al. (2022). Inorganic Se content was determined using the hydrochloric acid extraction method as described by Li et al. (2022). Organic Se content and translocation factor were determined using the following formulae: organic Se content = total Se content - inorganic Se content (Li et al., 2022); translocation factor (TF) = shoot Se content/root Se content (Rastmanesh et al., 2010).

### Statistical analysis

The data were analyzed using the SPSS 20.0.0 software (IBM, Chicago, IL, USA) with three repetitions. Data were normalized and subjected to a homogeneity test, followed by a one-way analysis of variance and a Duncan’s Multiple Range Test (*P* < 0.05). Moreover, the linear relationship between *A. argyi* water extract concentration, and root and shoot total Se content was analyzed using regression analysis. Pearson’s correlation was used to determine the relationships among biomass, total Se content, photosynthetic pigment content, antioxidant enzyme activity, soluble protein content, and Se metabolism-related enzyme activities. The grey relational analysis method was used to analyze biomass, root total Se content, photosynthetic pigment content, antioxidant enzyme activity, soluble protein content, and Se metabolism-related enzyme activity relationships with shoot total Se content, as described by Wang (2019b) and Ma et al. (2022).

## Results

### Peach biomass

**Figure 1:**
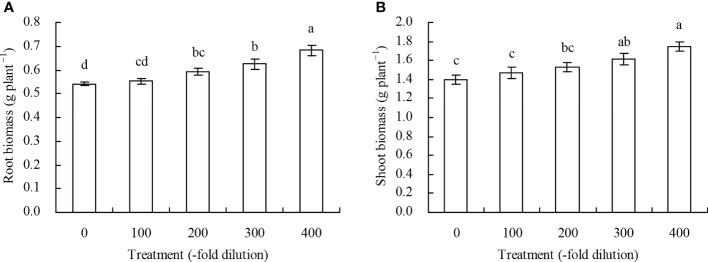
Biomass of peach seedlings. **(A)**: root biomass; **(B)**: shoot biomass. Values are means ± SD of three replicates. Different lowercase letters indicate significant differences among the treatments (Duncan’s Multiple Range Test, *P* < 0.05).

Both peach root and shoot biomass increased as the *A. argyi* water extract fold dilution increased (**Figures 1A, B**). Compared with the control, the 200-, 300-, and 400-fold dilutions of *A. argyi* water extract increased root biomass by 9.61%, 15.65%, and 26.34%, respectively, and the 300- and 400-fold dilutions increased shoot biomass by 15.94% and 25.44%, respectively. The other fold dilutions did not significantly affect root or shoot biomass.

### Photosynthetic pigment content in peach leaves

**Figure 2:**
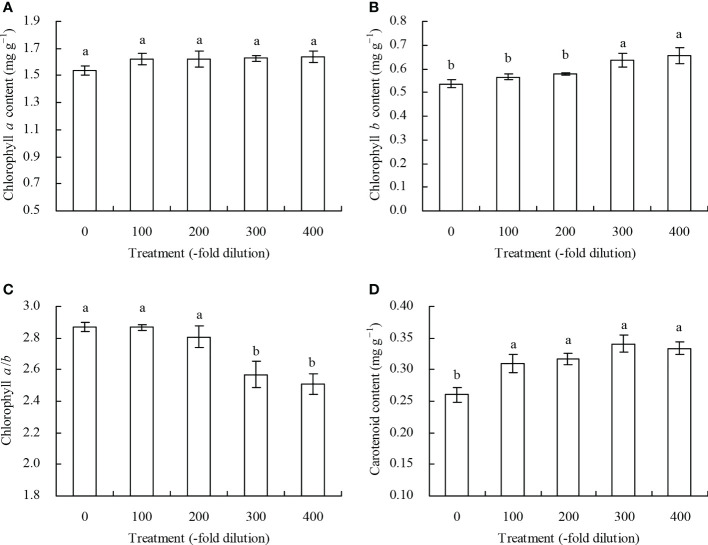
Photosynthetic pigment content in leaves of peach seedlings. **(A)**: chlorophyll *a* content; **(B)**: chlorophyll *b* content; **(C)**: chlorophyll *a*/*b*; **(D)**: carotenoid content. Values are means ± SD of three replicates. Different lowercase letters indicate significant differences among the treatments (Duncan’s Multiple Range Test, *P* < 0.05).

The different *A. argyi* water extract concentration did not significantly affect chlorophyll *a* content in the peach leaves (**Figure 2A**). The 300- and 400-fold dilution of *A. argyi* water extract increased peach leaf chlorophyll *b* content, but decreased chlorophyll *a*/*b*, compared with their respective controls, while the 100- and 200-fold dilutions did not significantly affect chlorophyll *b* content and chlorophyll *a*/*b* (**Figures 2B, C**). However, different *A. argyi* water extract concentrations increased peach leaf carotenoid content (**Figure 2D**).

### Peach leaf antioxidant enzyme activity and soluble protein content

**Figure 3:**
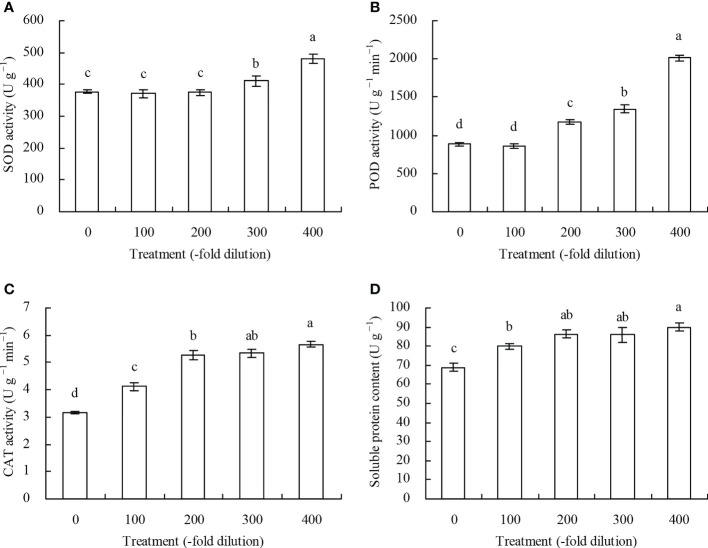
Antioxidant enzyme activity and soluble protein content of leaves of peach seedlings. **(A)**: SOD activity; **(B)**: POD activity; **(C)**: CAT activity; **(D)**: soluble protein content. Values are means ± SD of three replicates. Different lowercase letters indicate significant differences among the treatments (Duncan’s Multiple Range Test, *P* < 0.05).

The 100- and 200-fold dilutions of *A. argyi* water extract did not significantly affect peach leaf SOD activity, while the 300- and 400-fold dilutions increased SOD activity by 8.87% and 27.33%, respectively (**Figure 3A**). The 100-fold dilution did not significantly affect POD activity, while the 200-, 300-, and 400-fold dilutions increased POD activity by 32.28%, 51.97%, and 127.57%, respectively (**Figure 3B**). The different *A. argyi* water extract concentrations increased CAT activity and soluble protein content (**Figures 3C, D**).

### Different Se forms and their transport

**Figure 4:**
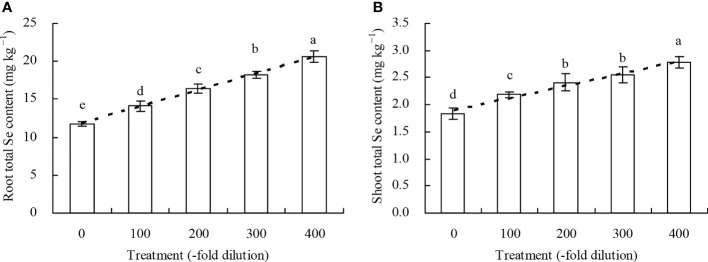
Total Se content in peach seedlings. **(A)**: root total Se content; **(B)**: shoot total Se content. Values are means ± SD of three replicates. Different lowercase letters indicate significant differences among the treatments (Duncan’s Multiple Range Test, *P* < 0.05).

**Figure 5:**
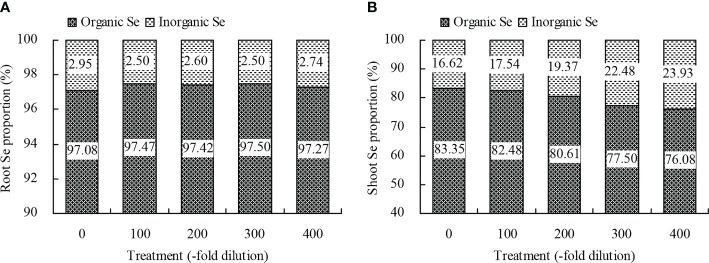
Proportions of organic Se and inorganic Se of peach seedlings. **(A)**: root Se proportion; **(B)**: shoot Se proportion.

**Figure 6:**
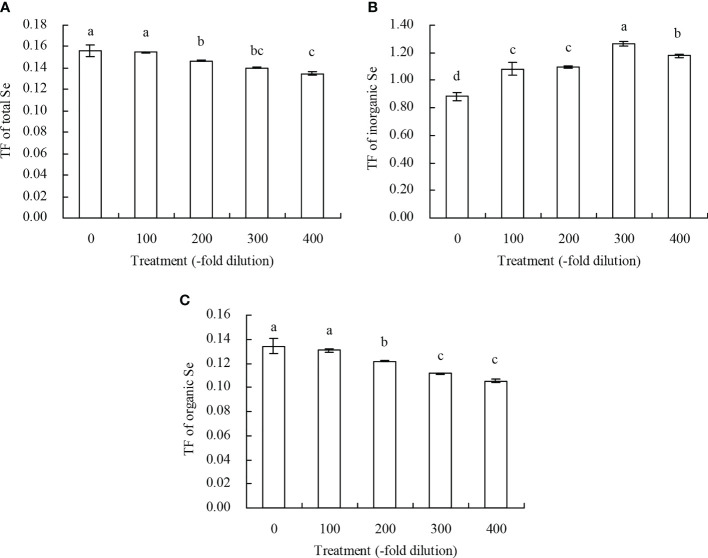
TF of Se of peach seedlings. **(A)**: TF of total Se; **(B)**: TF of inorganic Se; **(C)**: TF of organic Se. Values are means ± SD of three replicates. Different lowercase letters indicate significant differences among the treatments (Duncan’s Multiple Range Test, *P* < 0.05). Translocation factor (TF) = Se content in shoots/Se content in roots.

Peach root and shoot total Se content increased as the *A. argyi* water extract fold dilutions increased (**Figures 4A, B**). The 100-, 200-, 300-, and 400-fold dilutions increased root total Se content by 20.22%, 39.78%, 55.22%, and 75.72%, respectively, and increased shoot total Se content by 18.95%, 31.31%, 39.32%, and 51.59%, respectively. Moreover, regression analysis showed that *A. argyi* water extract concentration was linearly related to both the root total Se content (y = 0.022x + 11.848, R^2^ = 0.987, *P* = 0.000) and shoot total Se content (y = 0.002x + 1.900, R^2^ = 0.967, *P* = 0.000). Root and shoot inorganic Se content, and root and shoot organic Se content had similar fold dilution changes as *A. argyi* water extract increased, and were the same as total Se content (**Table 1**). The different *A. argyi* water extract concentrations decreased the root inorganic Se proportion, but increased the root organic Se proportion (**Figure 5A**). However, the different *A. argyi* water extract concentrations increased the shoot inorganic Se proportion, but decreased the shoot organic Se proportion (**Figure 5B**). The different *A. argyi* water extract concentrations decreased the TFs of total Se and organic Se to some extent (**Figures 6A, B**), while increasing the TF of inorganic Se, compared with their respective controls (**Figure 6C**).

**Table 1:** Inorganic Se and organic Se contents in peach seedlings.

Treatment (-fold dilution)	Root inorganic Se content(mg kg^−1^)	Shoot inorganic Se content(mg kg^−1^)	Root organic Se content(mg kg^−1^)	Shoot organic Se content(mg kg^−1^)
0	0.346 ± 0.005c	0.305 ± 0.013e	11.40 ± 0.48e	1.530 ± 0.002d
100	0.353 ± 0.010c	0.383 ± 0.005d	13.76 ± 0.24d	1.801 ± 0.051c
200	0.426 ± 0.016b	0.467 ± 0.014c	15.99 ± 0.50c	1.943 ± 0.054b
300	0.455 ± 0.018b	0.575 ± 0.015b	17.77 ± 0.58b	1.982 ± 0.054b
400	0.565 ± 0.020a	0.666 ± 0.018a	20.07 ± 0.80a	2.117 ± 0.059a

Values are means ± SD of three replicates. Different lowercase letters indicate significant differences among the treatments (Duncan’s Multiple Range Test, P < 0.05).

### Se metabolism-related enzyme activities of peach leaves

Peach leaf Se metabolism-related enzyme (ATPS, APR, SAT, and SMT) activities increased as the *A. argyi* water extract fold dilutions increased (**Table 2**). The 100-, 200-, 300-, and 400-fold dilutions of *A. argyi* water extract increased ATPS activity by 41.67%, 46.67%, 55.09%, and 64.26%, respectively, increased APR activity by 13.88%, 12.31%, 19.09%, and 23.21%, respectively, and increased SAT activity by 16.16%, 13.63%, 31.30%, and 36.05%, respectively. For SMT activity, only the 300- and 400-fold dilutions affected SMT activity, while the 100- and 200-fold dilutions had no significant effects.

**Table 2:** Se metabolism-related enzyme activity of leaves of peach seedlings.

Treatment (-fold dilution)	ATPS activity (U g^−1^)	APR activity (U g^−1^)	SAT activity (U g^−1^)	SMT activity (U g^−1^)
0	1.080 ± 0.041d	1.917 ± 0.016c	1.262 ± 0.018c	2.221 ± 0.082c
100	1.530 ± 0.037c	2.183 ± 0.087b	1.466 ± 0.022b	2.341 ± 0.043c
200	1.584 ± 0.026bc	2.153 ± 0.067b	1.434 ± 0.020b	2.369 ± 0.046c
300	1.675 ± 0.069ab	2.283 ± 0.029ab	1.657 ± 0.067a	2.668 ± 0.063b
400	1.774 ± 0.048a	2.362 ± 0.035a	1.717 ± 0.069a	2.886 ± 0.035a

Values are means ± SD of three replicates. Different lowercase letters indicate significant differences among the treatments (Duncan’s Multiple Range Test, P < 0.05).

### Correlation and grey relational analyses

**Figure 7:**
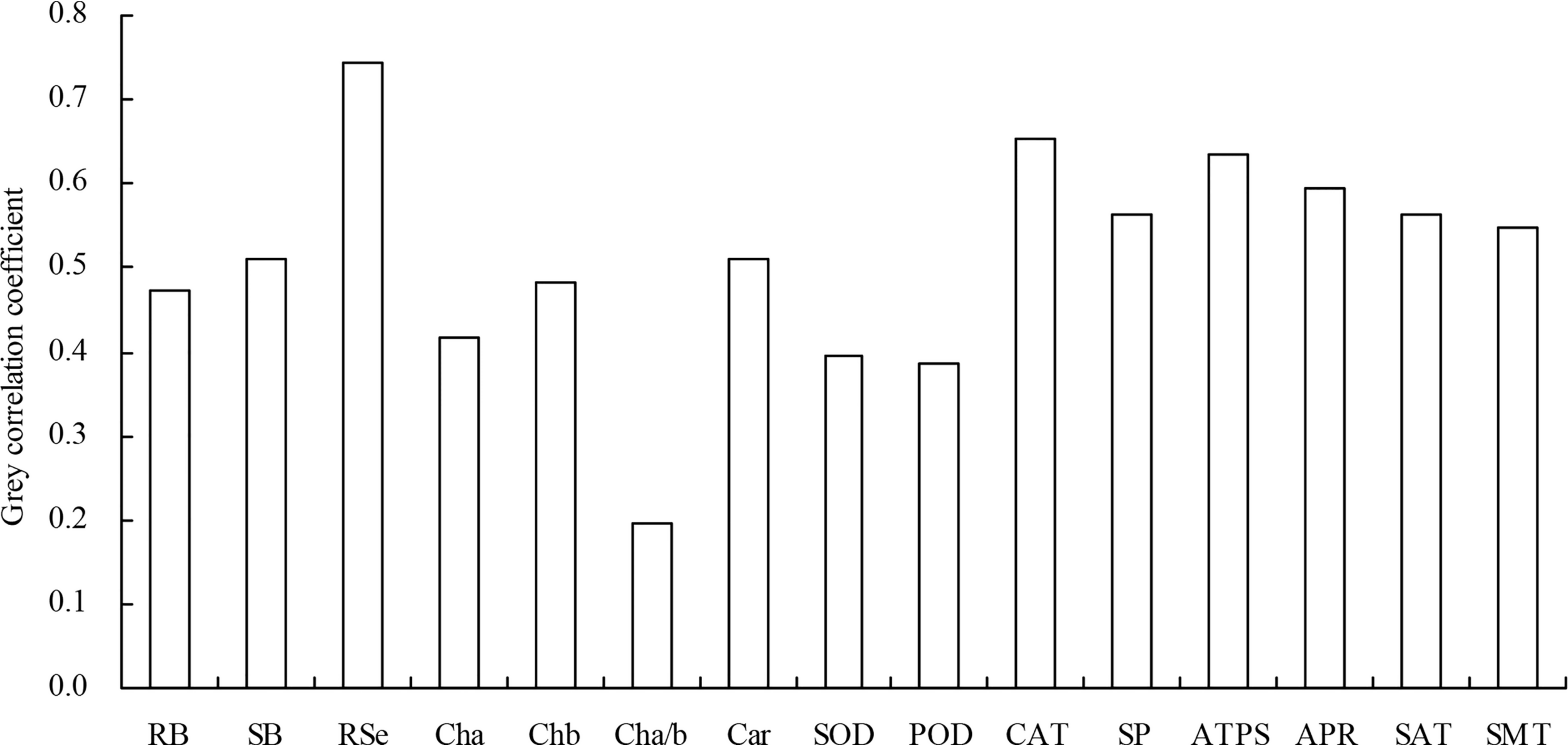
Grey correlation coefficient of the biomass, root total Se content, photosynthetic pigment content, antioxidant enzyme activity, soluble protein content, and Se metabolism-related enzyme activity with the shoot total Se content. RB, root biomass; SB, shoot biomass; RSe, root total Se content; Cha, chlorophyll *a* content; Chb, chlorophyll *b* content; Cha/b, chlorophyll *a*/*b*; Car, carotenoid content; SOD, SOD activity; POD, POD activity; CAT, CAT activity; SP, soluble protein content; ATPS, ATPS activity; APR, APR activity; SAT, SAT activity; SMT, SMT activity.

Highly significant (*P* < 0.01) positive, or significant (0.01 ≤ *P* < 0.05) positive correlations existed among root biomass, shoot biomass, root total Se content, shoot total Se content, chlorophyll *b* content, carotenoid content, SOD activity, POD activity, CAT activity, soluble protein content, ATPS activity, APR activity, SAT activity, and SMT activity (**Table 3**). Chlorophyll *a*/*b* was highly significantly (*P* < 0.01) and negatively correlated with all of the other indicators. The grey relational analysis showed that biomass, root total Se content, photosynthetic pigment content, antioxidant enzyme activity, soluble protein content, and Se metabolism-related enzyme activities were all correlated with shoot total Se content (**Figure 7**). The top three largest indicators of the grey correlation coefficients were root total Se content, CAT activity, and ATPS activity with shoot total Se content, and were also the top three closest relationships.

**Table 3:** Correlations among the biomass, total Se content, photosynthetic pigment content, antioxidant enzyme activity, soluble protein content, and Se metabolism-related enzyme activity.

Indicator	Root biomass	Shoot biomass	Root total Se content	Shoot total Se content	Chlorophyll *a* content	Chlorophyll *b* content	Chlorophyll *a*/*b*	Carotenoid content	SOD activity	POD activity	CAT activity	Soluble protein content	ATPS activity	APR activity	SAT activity	SMT activity
Root biomass																
Shoot biomass	0.954**															
Root total Se content	0.953**	0.929**														
Shoot total Se content	0.923**	0.911**	0.992**													
Chlorophyll *a* content	0.592*	0.759**	0.650**	0.697**												
Chlorophyll *b* content	0.905**	0.966**	0.899**	0.883**	0.744**											
Chlorophyll *a*/*b*	-0.908**	-0.906**	-0.879**	-0.839**	-0.515*	-0.955**										
Carotenoid content	0.725**	0.812**	0.854**	0.891**	0.868**	0.838**	-0.707**									
SOD activity	0.898**	0.902**	0.805**	0.744**	0.468	0.848**	-0.874**	0.530*								
POD activity	0.969**	0.928**	0.917**	0.873**	0.491	0.858**	-0.887**	0.624*	0.952**							
CAT activity	0.844**	0.859**	0.942**	0.964**	0.750**	0.840**	-0.764**	0.913**	0.617*	0.777**						
Soluble protein content	0.819**	0.864**	0.898**	0.927**	0.847**	0.838**	-0.711**	0.898**	0.616*	0.739**	0.958**					
ATPS activity	0.783**	0.832**	0.905**	0.947**	0.816**	0.826**	-0.714**	0.954**	0.585*	0.708**	0.936**	0.939**				
APR activity	0.839**	0.901**	0.900**	0.919**	0.850**	0.881**	-0.765**	0.929**	0.706**	0.757**	0.870**	0.907**	0.938**			
SAT activity	0.878**	0.872**	0.945**	0.942**	0.642**	0.883**	-0.863**	0.877**	0.757**	0.821**	0.848**	0.814**	0.903**	0.933**		
SMT activity	0.949**	0.923**	0.947**	0.913**	0.565*	0.908**	-0.921**	0.761**	0.906**	0.936**	0.799**	0.768**	0.800**	0.861**	0.949**	

N = 15. **: Correlation is significant at the 0.01 level (2-tailed test). *: Correlation is significant at the 0.05 level (2-tailed test).

## Discussion

Crop straw returning not only improves soil nutrient supplies (Zhang et al., 2022), but also releases allelochemicals during decay and decomposition, which has an allelopathy on the other crops (Qi et al., 2015). For crop seeds and seedlings, maize seeds treated with millet straw water extract promoted seedling root and shoot growth (Dong et al., 2016), while garlic straw water extract inhibited pepper and tomato seed germination rates (Liu et al., 2015). Cotton straw extract has an allelopathy promoting effect on lettuce seedling height and fresh weight (Liu et al., 2018b), while rape straw water extract increases rice seedling height and fresh weight (Zou et al., 2022). Moreover, *A. argyi* water extract inhibits radish and rape seedling growth, and *Artemisia lavandulifolia* water extract also inhibits *Tricium aestivum*, *Brassica chinensis*, *Glycine soja*, *Melilotus suaveolens*, *Setaria chondrachne*, and *Solidago decurrens* seedling growth (Zhang et al., 2006; Jiang et al., 2009). In this experiment, the 200-, 300-, and 400-fold dilutions of *A. argyi* water extract increased peach root biomass, while the 300- and 400-fold dilutions increased shoot biomass. The other *A. argyi* water extract concentrations did not significantly affect peach root or shoot biomass. These results are consistent with previous reports on straw water extracts from other plants (Dong et al., 2016; Liu et al., 2018b; Zou et al., 2022), but contrast with *A. argyi* water extract effects (Jiang et al., 2009). These results indicate that a low *A. argyi* water extract concentration (i.e., high fold dilution, such as 300- and 400-fold) could promote peach growth, while high concentrations (low fold dilution) had no effects, which may be related to *A. argyi* flavonoids (Li et al., 2016). This is because flavonoids can inhibit or promote plant growth at different concentrations (Zong et al., 2017). *A. argyi* water extract fold dilutions less than 100 may inhibit peach growth. Therefore, since *A. argyi* water extract inhibited the growth of other crops (Jiang et al., 2009), it may be related to high concentrations. This further suggests that a low *A. argyi* water extract concentration (high fold dilution) could promote peach growth.

Photosynthetic pigment content reflects plant photosynthetic capacity and affects plant growth (Tholen et al., 2007; Wang et al., 2013). Chlorophyll is related to the rate of photosynthesis and plays a crucial role in crop photosynthesis, and its content affects the absorption, transmission, and conversion of light energy by crop leaves (Scandalios, 1993; Jackson et al., 1996; Wang et al., 2008). Carotenoids are important light-absorbing auxiliary pigments in plant photosynthesis, which can absorb excess light energy and protect photosynthetic organelles (Lu et al., 2022). Different corn straw water extract concentrations are known to inhibit chlorophyll content in wheat seedlings, and this inhibitory effect is strengthened as the concentration increases (Zhen et al., 2015). However, pepper straw promotes the synthesis of photosynthetic pigments in watermelon seedlings (Wang and Hao, 2021), while *Artemisia anethifolia* water extract also promotes the synthesis of photosynthetic pigments in wheat (Guan, 2014). In this study, different *A. argyi* water extract concentrations did not significantly affect peach leaf chlorophyll *a* content, but increased carotenoid content. Only the 300- and 400-fold dilutions of *A. argyi* water extract increased chlorophyll *b* content, and decreased peach leaf chlorophyll *a*/*b*. These results differ from corn straw water extract effects on wheat seedlings (Zhen et al., 2015), but are, however, consistent with other studies (Wang and Hao, 2021; Guan, 2014). These results also indicate that *A. argyi* water extract could produce an allelopathic effect that promotes peach photosynthetic pigment synthesis to some extent, which may be related to *A. argyi* flavonoids content (Li et al., 2016). Therefore, *A. argyi* water extract had various effects on peach chlorophyll *a*, chlorophyll *b*, and carotenoid synthesis. Its mechanism therefore requires further study.

Antioxidant enzymes, including SOD, CAT, and POD, can remove excess reactive oxygen species (ROS) produced in plants (Fu et al., 2007; Li et al., 2007; Gan et al., 2010), thereby reducing or avoiding cell damage caused by ROS (Jiang et al., 2013). Soluble protein is an important osmotic regulating substance, which plays a protective role in cell substances and biofilms, and is one of the indicators of resistance (Shan, 2020). Previous studies showed that mustard straw decomposition treatment increased cowpea SOD, POD, and CAT activities (Chen et al., 2019), while maize straw water extract decreased wheat POD activity (Zhen et al., 2015). Additionally, wheat straw water extract increased the soluble protein content in *Salvia miltiorrhiza* and *Forsythia suspensa* (Hua and Li, 2019). In this study, only the 300- and 400-fold dilutions of *A. argyi* water extract increased peach SOD activity, while the 200-, 300-, and 400-fold dilutions increased POD activity. The different *A. argyi* water extract concentrations increased peach CAT activity and soluble protein content. These results are consistent with previous studies (Chen et al., 2019; Hua and Li, 2019), indicating that *A. argyi* water extract could improve peach resistance to some extent. Flavonoids can improve plant oxidative stress cause by the environment (Nakabayashi et al., 2014). Therefore, improving peach resistance may be related to *A. argyi* flavonoids (Li et al., 2016), which require further study.

Se is a trace element that promotes plant growth (Tang et al., 2022). Plants Se is mainly absorbed from the soil through the roots, and Se exists as various forms in the soil, such as selenate, selenite, elemental selenium, and organic selenium compounds (Wang et al., 2014). Selenite migrates with difficulty in plants, and most of the selenite absorbed by plant roots is directly assimilated into organic selenium within the roots, where it also accumulates (Li et al., 2008; Chen et al., 2014). Plant Se metabolism is a series of reactions completed by several related enzymes and proteins (Ma et al., 2017). Selenite is converted to selenide within the plant body after absorption, and selenide is then converted to selenocysteine by O-acetylserine lyase (OAS-TL) and acetylserine transferase (Wallenberg et al., 2010; Yarmolinsky et al., 2013). Increased selenium content in the environment can promote acetylserine transferase gene expression, which in turn increases selenocysteine conversion (Van Hoewyk et al., 2008). In this study, different *A. argyi* water extract concentrations increased the various peach Se forms (total, inorganic, and organic Se), Se metabolism-related enzyme (ATPS, APR, SAT, and SMT) activities, and inorganic Se TF to some extent, while decreasing total Se and organic Se TFs to some extent. The different *A. argyi* water extract concentrations decreased root inorganic Se, but increased the root organic Se, while increasing shoot inorganic Se and decreasing shoot organic Se. These results are consistent with a previous study (Liu et al., 2021), as well as other straw studies on crop nutrient uptake (Li, 2007; Zhang et al., 2015; Huang et al., 2017; Zhou et al., 2019), which indicate that *A. argyi* water extract could promote Se peach uptake. The reason may be that the allelopathic effects of *A. argyi* stimulated an increase in plant Se metabolism-related enzyme activities, as well as an increased Se resistance (Nakabayashi et al., 2014). Additionally, correlation and grey relational analyses showed that root total Se content, CAT activity, and ATPS activity were the three most closely associated factors with total shoot Se content, indicating that root total Se content, CAT activity, and ATPS activity significantly promoted peach Se uptake. However, their mechanisms require further study.

## Conclusion

The different *A. argyi* water extract concentrations increased peach carotenoid content, CAT activity, and soluble protein content, and only the 300- and 400-fold dilutions increased biomass, chlorophyll *a* content, SOD activity, and POD activity. The different *A. argyi* water extract concentrations also promoted peach total Se, inorganic Se, and organic content uptake to some extent. *A. argyi* water extract concentration exhibited a linear relationship with root and shoot total Se content by increasing Se metabolism-related enzyme activities. Notably, root total Se content, CAT activity, and ATPS activity were all closely associated with total shoot Se content. So, *A. argyi* water extract can promote the growth and Se uptake of peach seedlings. Future studies should focus on replicating these findings in peach fields to study the application effects of, and Se uptake in, peach fruits.

## Data availability statement

The original contributions presented in the study are included in the article/supplementary material. Further inquiries can be directed to the corresponding author.

## Author contributions

LL and ML conceived and designed the research and checked and revised the manuscript. JW, RH, QD, and ZW performed the experiments. XW and YT analyzed the data. LL prepared and wrote the manuscript. All authors contributed to this article and approved the submitted version.

## Conflict of interest

The authors declare that the research was conducted in the absence of any commercial or financial relationships that could be construed as a potential conflict of interest.

## Publisher’s note

All claims expressed in this article are solely those of the authors and do not necessarily represent those of their affiliated organizations, or those of the publisher, the editors and the reviewers. Any product that may be evaluated in this article, or claim that may be made by its manufacturer, is not guaranteed or endorsed by the publisher.
